# Supplementary material for: Toward reproducible models of sequence learning: replication and analysis of a modular spiking network with reward-based learning
Source: Front Integr Neurosci. 2023 Jun 15;17:935177. doi: 10.3389/fnint.2023.935177 (PMC10310927; doi:10.3389/fnint.2023.935177)
Supplement: Supplementary file 1 [file Data_Sheet_1.PDF]

Supplementary Material:

Towards reproducible models of sequence learning: replication and analysis of a modular spiking network with reward-based learning

1 SUPPLEMENTARY FIGURES

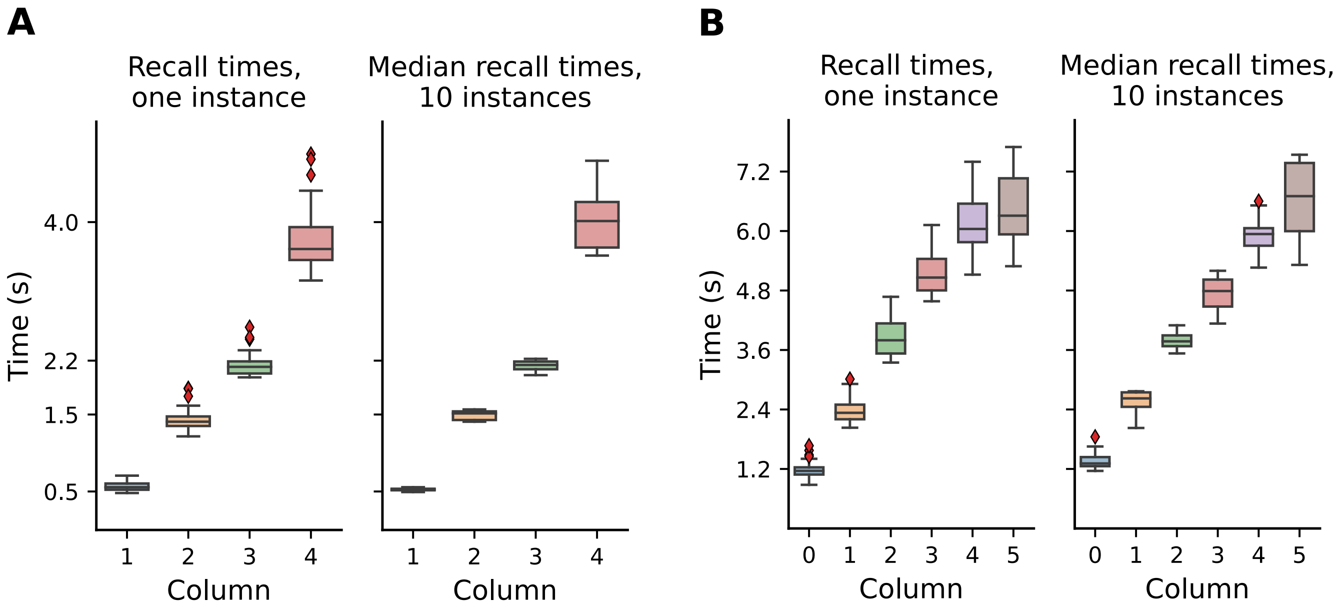

Figure S1. Fluctuations in learning and recall increase with sequence complexity and number of elements. (A) The network was trained on a sequence of four elements: $500, 1000, 700, 1800$ ms. Left: recall times for 30 trials after learning, for one network instance. Right: distribution of the median recall times over 10 network instances, with the median in each network calculated over 30 replay trials. **(B)** Same as (A), for a sequence of six elements with a duration of 1200 ms each.

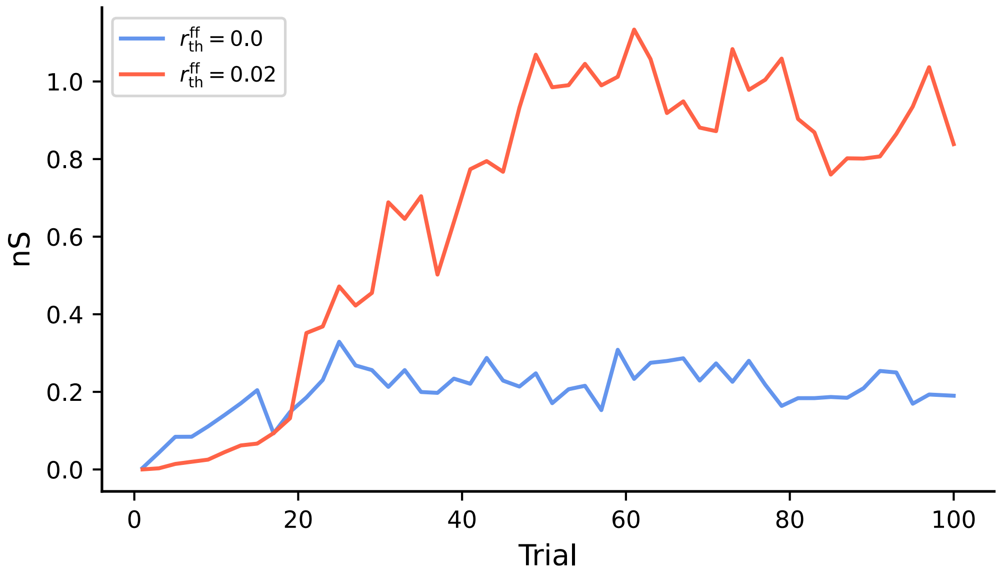

Figure S2. Hebbian threshold impacts learning convergence of cross-columnar connections. In the baseline network, learning succeeds even in the absence of a Hebbian threshold $r_{\mathrm{th}}^{\mathrm{ff}}$. While a non-zero threshold leads to larger synaptic weights after convergence, it also increases the variability between trials.

2 SUPPLEMENTARY TABLES

2.1 Baseline Model

**A: Model Summary**	
**Populations**	Multiple columns, each one composed of an excitatory Timer (layer L_5) and Messenger ($L_{2/3}$) population, with one inhibitory population in each layer
**Topology**	None
**Connectivity**	Sparse, random recurrent connectivity
**Neuron Model**	Leaky integrate-and-fire, fixed voltage threshold, fixed absolute refractory time, no adaptation
**Synapse Model**	Conductance-based, exponentially decaying PSCs, static and plastic synaptic weights, fixed delays
**Plasticity**	Reward-based plasticity, short-term adaptation
**Input**	Stochastic background current and inhomogeneous Poisson spikes onto stimulus-specific T and I_{T}
**Measurements**	Spiking activity

**B: Populations**		
**Name**	**Elements**	**Size**
$T^i, I_{\mathrm{T}}^i, M^i, I_{\mathrm{M}}^i$ in column C_i	LIF neuron	100

**C: Neuron Models**	
**Name**	Leaky integrate-and-fire (LIF) neuron
**Subthreshold Dynamics**	if $(t > t^* + \tau_{\mathrm{ref}})$<br>$C_{\mathrm{m}} \frac{dV_i}{dt} = g_{\mathrm{L}} \left(V_{\mathrm{rest}} - V_i(t) \right) + I_i^{\mathrm{E}}(t) + I_i^{\mathrm{I}}(t) + \xi(t)$<br>else<br>$V(t) = V_{\mathrm{reset}}$<br>$I_{\mathrm{ij}}^{\mathrm{syn}}(t) = g_{\mathrm{ij}}^{\mathrm{syn}}(E_{\mathrm{syn}} - V_i(t))$
**Spiking**	If $V(t-) < V_{\mathrm{th}}$ OR $V(t+) \geq V_{\mathrm{th}}$<br>1. set $t^* = t$ 2. emit spike with time stamp t^*

**D: Synapse Models**	
**Synaptic trace**	$\frac{d\mathrm{s_i}}{dt} = -\frac{\mathrm{s_i}}{\tau_{\mathrm{s}}} + \rho \left(1 - \mathrm{s_i} \right) \sum_{\mathrm{k}} \delta \left(\mathrm{t} - \mathrm{t_k^i} \right)$
**Name**	Reward-based, with separate LTP and LTD eligibility traces
**Trace update rule**	$\tau^a \frac{dT_{ij}^a(t)}{dt} = -T_{ij}^a(t) + \eta_{(\mathrm{ff})}^a H_{ij}(t) \left(T_{\max}^a - T_{ij}^a(t) \right), a \in \{p, d\}$<br>$H_{ij}(t) = \begin{cases} r_i(t) r_j(t) & \text{if } r_i(t) r_j(t) > r_{\mathrm{th}}^{(\mathrm{ff})} \\ 0 & \text{otherwise} \end{cases}$
**Online update rule**	$\frac{dw_{ij}}{dt} = \eta_{(\mathrm{ff})} R(t) \left(T_{ij}^p - T_{ij}^d \right)$<br>$R(t) = \delta(t - t_{\mathrm{reward}} - d_{\mathrm{reward}})$

**E: Input**		
**Type**	**Target**	**Description**
poisson_generator	T^i and I_{T}^i in C_i	Total rate ν_{in} for a duration of $50\ \mathrm{ms}$

**F: Measurements**
Spiking activity

Table S1. Tabular description of network model after Nordlie et al. (2009).

A: Populations			
**Name**	**Value**	**Source**	**Description**
N	100	paper	Population size of every population, excitatory and inhibitory

B: Connectivity			
**Name**	**Value**	**Source**	**Description**
d	1 ms	code	Synaptic transmission delay
φ	0.26	code*	Connection probability for all populations
w_{in}	100 nS	code	Synaptic strength of input connections
$w_{T\to M}$	0.2 nS	code*	Intracolumnar T to M excitatory synaptic strength
$w_{I_{\mathrm{T}}\to M}$	70 nS $^{\circledast}$	code*	Inhibitory synaptic strength from I_{T} in L_5 to M
$w_{I^i_{\mathrm{T}}\to T^j}$	100 nS $^{\circledast}$	code*	Inhibitory synaptic strength from I^i_{T} in C_i to T^j in C_j
$w_{I^i_{\mathrm{M}}\to M^j}$	100 nS $^{\circledast}$	code*	Inhibitory synaptic strength from I^i_{M} in C_i to M^j in C_j
$w_{T\to I_{\mathrm{T}}}$	0.2 nS $^{\circledast}$	code*	Intracolumnar T to I_{T} excitatory synaptic strength
$w_{M\to I_{\mathrm{M}}}$	1 nS $^{\circledast}$	code*	Intracolumnar M to I_{M} excitatory synaptic strength

B: Neuron Model			
**Name**	**Value**	**Source**	**Description**
C_{m}	200 pF	paper	Membrane capacitance
τ_{m}	10 ms	paper	Membrane time constant
g_{L}	10 nS	paper	Leak conductance
E_L	-60 mV	paper	Resting membrane potential
$V^{\mathrm{E}}_{\mathrm{th}}$	-55 mV	paper	Spiking threshold for excitatory neurons
$V^{\mathrm{I}}_{\mathrm{th}}$	-50 mV	code*	Spiking threshold for inhibitory neurons
V_{reset}	-60 mV	code*	Reset potential
τ_{ref}	3 ms	code*	Absolute refractory period
σ_ξ	100	code*	Standard deviation of Gaussian white noise
ν_{in}	30 Hz	code*	Rate of Poisson stimulus input

C: Synapse Model			
**Name**	**Value**	**Source**	**Description**
E_{E}	0 mV	paper	Excitatory reversal potential
E_{I}	-70 mV	paper	Inhibitory reversal potential
$\tau^{\mathrm{exc,inp}}_{\mathrm{syn}}$	10 ms	code*	Excitatory synaptic time constant of the input connections
$\tau^{\mathrm{exc}}_{\mathrm{syn}}$	80 ms	paper	Excitatory synaptic time constant
$\tau^{\mathrm{inh}}_{\mathrm{syn}}$	10 ms	paper	Inhibitory synaptic time constant
ρ	$1/7$	paper	Fractional change of synaptic activation

Table S2. Tabular description of the neuron, synapse and connectivity parameters. Parameters marked with $^{\circledast}$ were additionally jittered with a randomly drawn value from $\mathcal{N}(0, 0.1)$. Parameters marked with * had different values in the code than reported in the paper.

A: Learning Parameters			
**Name**	**Value**	**Source**	**Description**
τ^p	2000 ms	paper	LTP eligibility trace time constant (intracolumnar connections)
τ^d	1000 ms	paper	LTD eligibility trace time constant (intracolumnar connections)
τ^p_{ff}	200 ms	paper	LTP eligibility trace time constant (cross-columnar connections)
τ^d_{ff}	800 ms	paper	LTD eligibility trace time constant (cross-columnar connections)
T^p_{max}	0.0033	code*	Saturation level of LTP trace (intracolumnar connections)
T^d_{max}	0.00345	code*	Saturation level of LTD trace (intracolumnar connections)
$T^{p,\text{ff}}_{\text{max}}$	0.0034	code*	Saturation level of LTP trace (cross-columnar connections)
$T^{d,\text{ff}}_{\text{max}}$	0.00345	code*	Saturation level of LTD trace (cross-columnar connections)
η^p	45×3500 ms^{-1}	code*	Activation rate of LTP trace (intracolumnar connections)
η^d	25×3500 ms^{-1}	code*	Activation rate of LTD trace (intracolumnar connections)
η^p_{ff}	20×3500 ms^{-1}	code*	Activation rate of LTP trace (cross-columnar connections)
η^d_{ff}	15×3500 ms^{-1}	code*	Activation rate of LTD trace (cross-columnar connections)
r_{th}	10 Hz	code*	Hebbian activation threshold (recurrent connections)
$r^{\text{ff}}_{\text{th}}$	20 Hz	code*	Hebbian activation threshold (feedforward connections)
η	0.16 ms^{-1}	code*	Learning rate $T \to T$ connections
η	20 ms^{-1}	code*	Learning rate $M \to T$ connections
T_{reward}	25 ms	paper	Duration of neuromodulator presentation upon change in stimulus
T_{tr}	25 ms	paper	Duration of refractory period for traces following neuromodulator presentation
d_{reward}	25 ms	paper	Reward delay

Table S3. Tabular description of learning parameters. Parameters marked with * had different values in the code than reported in the paper.

2.2 Scaled model

A: Parameters for standard scaling		
**Name**	**Value**	**Description**
N'	400	Number of neurons in each population (scaled)
$w'_{T\to M}$	$w_{T\to M}/2$	Intracolumnar T to M excitatory synaptic strength
$w'_{I_{\mathrm{T}}\to M}$	$w_{I_{\mathrm{T}}\to M}/2$ ⊛	Inhibitory synaptic strength from I_{T} in L_5 to M
$w'_{I^i_{\mathrm{T}}\to T^j}$	$w_{I^i_{\mathrm{T}}\to T^j}/2$ ⊛	Inhibitory synaptic strength from I^i_{T} in C_i to T^j in C_j
$w'_{I^i_{\mathrm{M}}\to M^j}$	$w_{I^i_{\mathrm{M}}\to M^j}/2$ ⊛	Inhibitory synaptic strength from I^i_{M} in C_i to M^j in C_j
$w'_{T\to I_{\mathrm{T}}}$	$w_{T\to I_{\mathrm{T}}}/2$ ⊛	Intracolumnar T to I_{T} excitatory synaptic strength
$w'_{M\to I_{\mathrm{M}}}$	$w_{M\to I_{\mathrm{M}}}/2$ ⊛	Intracolumnar M to I_{M} excitatory synaptic strength
**B: Parameters for manually tuned scaling**		
**Name**	**Value**	**Description**
N''	400	Number of neurons in each population (scaled)
$w''_{T\to M}$	$w'_{T\to M}\cdot 1.2$	Intracolumnar T to M excitatory synaptic strength
$w''_{I_{\mathrm{T}}\to M}$	$w'_{I_{\mathrm{T}}\to M}\cdot 2$ ⊛	Inhibitory synaptic strength from I_{T} in L_5 to M
$w''_{I^i_{\mathrm{T}}\to T^j}$	$w'_{I^i_{\mathrm{T}}\to T^j}\cdot 0.02$ ⊛	Inhibitory synaptic strength from I^i_{T} in C_i to T^j in C_j
$w''_{I^i_{\mathrm{M}}\to M^j}$	$w'_{I^i_{\mathrm{M}}\to M^j}\cdot 2$ ⊛	Inhibitory synaptic strength from I^i_{M} in C_i to M^j in C_j
$w''_{T\to I_{\mathrm{T}}}$	$w'_{T\to I_{\mathrm{T}}}\cdot 0.02$ ⊛	Intracolumnar T to I_{T} excitatory synaptic strength
$w''_{M\to I_{\mathrm{M}}}$	$w'_{M\to I_{\mathrm{M}}}\cdot 2$ ⊛	Intracolumnar M to I_{M} excitatory synaptic strength
σ''_ξ	$\sigma_\xi/2$	Standard deviation of Gaussian white noise

Table S4. Tabular description of the modified parameters in the scaled network models. For the standard scaling, the values are obtained by applying a scaling factor of $1/\sqrt{N'/N}$ to the original values (see Methods). Parameters marked with ⊛ were additionally jittered with a randomly drawn value from $\mathcal{N}(0, 0.1)$.

2.3 Alternative model with local inhibition

A: Parameters for Network with Local Inhibition		
**Name**	**Value**	**Description**
N	100	Number of neurons in each population (as in baseline model)
$w_{T \to M}$	0.2 nS	Intracolumnar T to M excitatory synaptic strength
$w_{I_\mathrm{T} \to T}$	70 nS$^{\circledast}$	Inhibitory synaptic strength from I_T in L_5 to T
$w_{I_\mathrm{M} \to M}$	70 nS$^{\circledast}$	Inhibitory synaptic strength from I_M in $L_{2/3}$ to M
$w_{T \to I_\mathrm{M}}$	0.2 nS$^{\circledast}$	Excitatory synaptic strength from T to I_M
$w_{T^i \to I_\mathrm{T}^j}$	0.2 nS$^{\circledast}$	Excitatory synaptic strength from T in column C_i to I_T in $C_j, i \neq j$
$w_{M^i \to I_\mathrm{M}^j}$	0.5 nS$^{\circledast}$	Excitatory synaptic strength from M in column C_i to I_M in $C_j, i \neq j$

Table S5. Tabular description of the modified parameters in the model with rewired local inhibition. Parameters marked with $^{\circledast}$ were additionally jittered with a randomly drawn value from $\mathcal{N}(0, 0.1)$.

REFERENCES

Nordlie, E., Gewaltig, M.-O., and Plesser, H. E. (2009). Towards reproducible descriptions of neuronal network models. *PLoS Computational Biology* 5, e1000456. doi:10.1371/journal.pcbi.1000456
